# THEORETICALLY BASED FACTORS ASSOCIATED WITH STROKE FAMILY CAREGIVER HEALTH

**DOI:** 10.1093/geroni/igad104.2978

**Published:** 2023-12-21

**Authors:** Cleopatra Kum, Holly Jones, Elaine Miller, Natalie Kreitzer, Tamilyn Bakas

**Affiliations:** University of Cincinnati, Cincinnati, Ohio, United States; The Ohio State University, Columbus, Ohio, United States; University of Cincinnati, Cincinnati, Ohio, United States; University of Cincinnati College of Medicine, Cincinnati, Ohio, United States; University of Cincinnati, Cincinnati, Ohio, United States

## Abstract

**Background:**

Most stroke survivors are cared for at home by family members, many of whom experience depressive symptoms and life changes as a result of providing care. While many studies have explored the factors associated with depressive symptoms and quality of life in family caregivers, few have focused on caregiver health. Purpose: To determine theoretically based factors associated with unhealthy days in stroke family caregivers.

**Methods:**

A conceptual model derived from Lazarus’ theory of stress and coping was used to guide analysis. Secondary data analysis was conducted using baseline data from a large randomized controlled clinical trial testing the Telephone Assessment and Skill-building Kit (TASK II) program with 254 family caregivers of stroke survivors within 2 weeks of discharge from acute care and rehabilitation settings in participating Midwestern facilities. Data were analyzed using multiple regression with unhealthy days as the dependent variable, and theoretically based factors (i.e., caregiver task difficulty, level of optimism, threat appraisal, depressive symptoms, and life changes) as independent variables. All scales demonstrated evidence of internal consistency reliability with Cronbach alpha scores above .70.

**Results:**

Caregivers were mostly female (78%), white (71%), spouses (47%) or adult children (30%). Caregivers reported 9 unhealthy days on average within the past month. Unhealthy days were significantly correlated with caregiver task difficulty (r=.32), level of optimism (r=-.29), threat appraisal (r=.44), depressive symptoms (r=.60), and life changes (r=-.40) (p<.05). 37.8% variance in unhealthy days was explained, with depressive symptoms being the only significant individual predictor due to shared variance.

